# Supplementary material for: Autophagy across tissues of aging mice
Source: PLoS One. 2025 Jun 4;20(6):e0325505. doi: 10.1371/journal.pone.0325505 (PMC12136466; doi:10.1371/journal.pone.0325505)
Supplement: Attachment — Submitted filename: Response to Reviewers.pdf [file pone.0325505.s011.pdf]

Response to reviewers:

We have now revised the paper in response to the editor/reviewer #2’s comment: **“it would be important to provide quantifications of the WB for autophagic proteins and cathepsin D”.**

We have provided quantifications of protein levels from immunoblots in Supplementary Figure 4A and B as heatmaps in Supplementary Figure 4C and D.
